# Supplementary material for: Aging and episodic memory specificity: Evidence challenging a domain-general pattern separation decline
Source: PLoS One. 2025 Nov 24;20(11):e0336045. doi: 10.1371/journal.pone.0336045 (PMC12643304; doi:10.1371/journal.pone.0336045)
Supplement: S1 File — (DOCX) [file pone.0336045.s001.docx]

**Supplementary Materials**

**Supplementary Methods**

**Similarity mixed model**

A generalized linear mixed-effects model was fit using the “glmer” function from the “lme4” package in R for each similarity metric. We used this model to predict the response variable “accuracy” from the VDT, based on several predictors, described in Table S1. The model equation provides the example for Meadows mean similarity as follows:

$$meadows.model\leftarrow glmer\left( accuracy \sim meadows.mean.sc*age.ec+meadows.mean.sc*shipley.std.sc+mean.length.sc+mean.conc.sc+mean.freq.sc+\left( meadows.mean.sc+mean.length.sc+mean.conc.sc+mean.freq.sc \right|subject \right),$$

$$family=binomial,$$

$$data=similarity\_summary\_scaled$$

**S1 Table. Model terms in the meadows mean similarity mixed model**

| Model Term | Definition |
| --- | --- |
| Meadows.mean.sc | Mean similarity rating between new test and studied words, normalized |
| Age.ec | Age group, effect-coded |
| Shipley.std.sc | Standardized Shipley-II Vocabulary scores, normalized |
| Mean.length.sc | Mean length of word, normalized |
| Mean.conc.sc | Mean concreteness/abstractness of word, normalized |
| Mean.freq.sc | Mean frequency index of word, normalized |

**S2 Table. Mixed model outcomes for different similarity metrics**

| Similarity Metric | Predictor Variable | Mean Model | | | | Max Model | | | |
| --- | --- | --- | --- | --- | --- | --- | --- | --- | --- |
| WordNet: Leacock & Chodorow |  | B | SE | *p* | *AIC* | B | SE | *p* | *AIC* |
|  | Similarity | -0.04 | 0.03 | 0.14 | 11592.129 | -0.02 | 0.02 | *0.23* |  |
|  | Age | 0.32 | 0.08 | **<0.0001** |  | 0.32 | 0.07 | **<0.0001** |  |
|  | Shipley | 0.10 | 0.07 | 0.2 |  | 0.10 | 0.08 | 0.19 |  |
|  | Mean Length | -0.26 | 0.03 | **<0.0001** |  | -0.26 | 0.03 | **<0.0001** |  |
|  | Mean Concreteness | 0.22 | 0.03 | **<0.0001** |  | 0.23 | 0.03 | **<0.0001** | 11601.58 |
|  | Mean Frequency | -0.06 | 0.03 | **0.03** |  | -0.06 | 0.03 | **0.03** |  |
|  | Sim: Age | -0.03 | 0.03 | 0.20 |  | 0.001 | 0.02 | 0.91 |  |
|  | Sim: Shipley | -0.011 | 0.03 | 0.68 |  | -0.02 | 0.02 | 0.25 |  |
| Word2Vec | Similarity | -0.009 | 0.03 | 0.76 | 11595.894 | -0.1 | 0.03 | **<0.0001** | 11587.947 |
|  | Age | 0.33 | 0.08 | **<0.0001** |  | 0.32 | 0.08 | **<0.0001** |  |
|  | Shipley | 0.09 | 0.03 | 0.20 |  | 0.10 | 0.08 | 0.18 |  |
|  | Mean Length | -0.26 | 0.03 | **<0.0001** |  | -0.26 | 0.03 | **<0.0001** |  |
|  | Mean Concreteness | 0.23 | 0.03 | **<0.0001** |  | 0.21 | 0.03 | **<0.0001** |  |
|  | Mean Frequency | -0.06 | 0.03 | **0.03** |  | -0.06 | 0.03 | **0.04** |  |
|  | Sim: Age | 0.05 | 0.02 | **0.02** |  | 0.01 | 0.02 | 0.55 |  |
|  | Sim: Shipley | -0.02 | 0.02 | 0.3 |  | -0.03 | 0.02 | 0.27 |  |
| Meadows | Similarity | -0.13 | 0.03 | **<0.0001** | 11560.148 | -0.03 | 0.02 | 0.16 |  |
|  | Age | 0.33 | 0.08 | **<0.0001** |  | 0.32 | 0.08 | **<0.0001** |  |
|  | Shipley | 0.11 | 0.08 | 0.17 |  | 0.10 | 0.08 | 0.18 |  |
|  | Mean Length | -0.24 | 0.03 | **<0.0001** |  | -0.26 | 0.03 | **<0.0001** | 11603.689 |
|  | Mean Concreteness | 0.20 | 0.03 | **<0.0001** |  | 0.24 | 0.03 | **<0.0001** |  |
|  | Mean Frequency | -0.04 | 0.03 | 0.18 |  | -0.06 | 0.03 | **0.03** |  |
|  | Sim: Age | 0.04 | 0.03 | 0.20 |  | 0.003 | 0.02 | 0.89 |  |
|  | Sim: Shipley | 0.01 | 0.03 | 0.60 |  | -0.003 | 0.02 | 0.92 |  |

We identified the best fitting model by selecting the one that yielded the smallest AIC value, which has the least information loss relative to the true model [1]. In our set of models, the Meadows Mean Model has the lowest AIC. Note that our key outcome (interaction between age and similarity) was only significant in 1 out of the 6 models.

**Prolific Sample**

We recruited 472 adults (*M =* 48.3, *SD =* 20.4*)* on Prolific (https://www.prolific.com/). This sample size ensured there would be a minimum number of ratings required for unique pairings between 324 words. Participants were recruited from Canada and the United States, were between the ages of 18-90, were fluent in English, and had no history of head injury or psychiatric, neurological, or learning disorders. In this sample, 74.2% of participants reported to be White, 11.4% Asian, 7.6% Black, 4.2% Mixed, and 2.5% Other. Although all participants were fluent in English, 88.1% reported English as their native language, while the remaining 11.9% reported a different first language. Years of education and Shipley-II Vocabulary scores were not collected for this sample.

**Task Design & Analysis**

Participants remotely performed a spatial multi-arrangement task on Meadows Research [2] in which 20 words were displayed on the computer screen, in random order, around a circular arena. Participants were instructed to arrange the nouns based on the similarity of their meaning by dragging and dropping the nouns one by one onto the circle. They were instructed to place similar words more closely together and less similar ones further apart; the relative distances between the words reflected the degree of similarity. Words that were placed closer together in this first trial were subsampled in subsequent trials and arranged again in a circular arena with fewer words, which ensured a higher signal-to-noise ratio. The representational dissimilarity matrix was updated after each trial and the evidence from consecutive arrangements were combined to produce the final pairwise dissimilarities for the entire stimulus set (for analysis details see [3]).

**S3 Table. Demographics of Online Sample**

| Sex (F:M) | Mean age(SD) | Age range |
| --- | --- | --- |
| 237:235 | 48.3(20.4) | 18-82 |

**Process dissociation procedure formulae**

Behavioural pattern completion was indexed using automatic estimates derived from the process dissociation procedure (PDP). According to PDP, correct responding in the inclusion task — completing the stem with a previously studied item — can be accomplished through controlled recollection (C), automatic memory reactivation (A), or both. If automatic influences and recollection are independent processes, the probability of a correct response is:

𝐼𝑛𝑐𝑙𝑢𝑠𝑖𝑜𝑛 = C + 𝐴 − CA

For the exclusion task — completing the stem with a word that has not appeared in the context of the study — recollection and automatic processes act in opposition. An earlier studied word can be mistakenly given as a completion word if the automatic process is not opposed by the cognitive control process. Accordingly, the probability of answering an exclusion stem with an earlier studied word is:

𝐸𝑥𝑐𝑙𝑢𝑠𝑖𝑜𝑛 = 𝐴 × *(1-C)*

Using linear algebra, automatic processes (A) can be isolated from controlled ones (C):

C = 𝐼𝑛𝑐𝑙𝑢𝑠𝑖𝑜𝑛 − 𝐸𝑥𝑐𝑙𝑢𝑠𝑖𝑜𝑛

𝐴 = 𝐸𝑥𝑐𝑙𝑢𝑠𝑖𝑜𝑛 / *(1-C)*

**Neuropsychological Tests for Older Adults**

A battery of neuropsychological tests were administered to older adults after they completed experimental tasks. The order of the tests was as follows: Digit Span, WMS-IV Verbal Paired Associates (adult version), Trail Making Test, Judgment of Line Orientation, Golden Stroop, Object Decision subtest of the VOSP, Doors and Names subtests of the Doors and People Test, Hayling Test, Verbal Fluency: FAS and Animals, Boston Naming Test (Short Version), and questionnaires.

Please note that the outcomes of these tests are not reported in the current manuscript, as our focus was on age group comparisons and neuropsychological data were not collected for the younger adult group. These data will be reported in future manuscripts.

**Supplementary Note 1**

**Exploratory analyses for the Verbal Completion Task**

We tested whether results differed within a sub-sample of participants who reported English as their first language. Specifically, we conducted critical age-group comparisons within this sub-sample (younger n=29; older n=50). Replicating results observed in the full sample, older adults were less likely to engage in both controlled (t(77) = -3.05, *p*=0.003, *d*=0.71), and automatic processes (t(77) = -3.23, *p*=0.001, *d*=0.75), compared to younger adults (S1 Fig). When accounting for performance on the Shipley-II, reduced automatic estimates was again noted in the older adult group (β=0.11, SE=0.03, p=0.001).

**Supplementary Note 2**

**Exploratory analyses of the influence of the number of common associates of each studied word on automatic estimates derived from the VCT**

We hypothesized that studied words with stems that had a greater number of possible completions would have heightened competition and interference at retrieval, resulting in reduced automatic reactivation of the studied word. For example, the stem “VEL” 2 common associates) may be more likely to be completed with the studied word “VELOCITY”, as opposed to the stem “CON” (427 common associates) to be completed with the studied word as “CONFERENCE.” We identified the number of common associates for each stem using Merriam-Webster’s Word Finder function [4]. We then median split each participant’s trial by the number of common associates and computed automatic estimates for low versus high numbers of possible completions. We then ran a linear model and found that a higher number of associates led to lower automatic estimates (β= -0.23, SE=0.04, p<0.0001); as predicted, when words had stems with more associates, it impeded the automatic reactivation of the studied word. There was a trending interaction between age and the number of associates (β= -0.1, SE=0.05, p=0.06), largely driven by the greater automatic estimates for words with lower numbers of associates in young adults (S2 Fig). Ultimately, while older adults had reduced automatic estimates compared to younger adults, this effect was not driven by the number of associates that may have competed at retrieval, further supporting a dedifferentiation account.

**Supplementary Note 3**

**Exploratory analyses testing the relationship between VCT controlled estimates and VDT slopes**

We were interested in seeing if there was a correlation between controlled estimates derived from the VCT, which engageexecutive control at retrieval , and VDT slopes, our index of behavioural pattern separation. We hypothesized that they would share a negative relationship, in that greater controlled processes should facilitate behavioural pattern separation. Contrary to our hypothesis, we did not find a relationship between these variables within younger (R= -0.023, p=0.86) nor older (R= -0.11, p=0.38) adult groups (S3 Fig).

**Supplementary Note 4**

**Chronological age as a continuous covariate for discrimination tasks**

To account for the wide age range in our older adult sample (range: 55-90), we conducted supplementary analyses of performance on the discrimination tasks, in which we included age as a continuous covariate mean-centered within each group (i.e., within-group age). Across all models and tasks, the inclusion of within-group age did not significantly influence the results, indicating that the observed group differences were not driven by age variability within the age groups.

**VDT**

A generalized linear mixed-effects model (binomial) was used to predict trial-level accuracy across new trials (i.e. items not shown at study) based on semantic similarity, age group, vocabulary, and lexical covariates, including subject-level random effects. There was a significant main effect of similarity (β = –0.17, SE = 0.04, z = –4.38, p < .001), with greater similarity associated with reduced accuracy. Younger adults showed significantly higher accuracy than older adults (β = 0.67, SE = 0.16, z = 4.24, p < .001). However, there was no significant interaction between similarity and age group (β = 0.07, p = .19), nor between similarity and vocabulary (β = 0.015, p = .60). Within-group age was not a significant predictor (β = 0.006, p = .61).

**MST**

A two (Age Group: Younger, Older) × two (Trial Type: Old, Similar) ANCOVA was conducted on MST discrimination scores (*d*’_a_), controlling for within-group age. There was a significant main effect of trial type (F(1, 135) = 73.25, p < .001), and a significant Age Group × Trial Type interaction (F(1, 135) = 31.92, p < .001). The effect of within-group age (F(1, 135) = 0.50, p = .48) was not significant.

**Supplementary Note 5**

**Age and automatic estimates in the older adult sample**

Age-related declines in automatic memory reactivation are inconsistently observed in the literature (S5 Table). Accordingly, we explored two possible explanations for divergent findings. First, we assessed the role of age. There was no significant correlation between age and automatic estimates within the older adult group (S5 Table; *r*(64) = -0.18, *p=.*14). Qualitatively, we observed a slight inflection point around the age of 70 followed by a slight gradual decline in automatic processing (S5 Fig). Furthermore, when we conducted supplementary analyses incorporating age as a continuous covariate centered within each group (i.e., within-group age), there was no significant effect of within-group age (β = –0.002, p = .26). These results suggest that the age range of our older adult group may not explain discrepancies across the literature. Indeed, sampled ages were similar across studies with discrepant findings (see S5 Table for summary of findings). Rather, a key difference between the studies may be related to the encoding task and its associated levels of processing: Jennings & Jacoby [5] and Rybash, Santoro, & Hoyer [6] required subjects to actively engage with the stimuli through creative tasks and cognitive processing techniques, contrasting with less generative and more passive encoding tasks in Toth & Parks [7] and Parks, Jacoby, & Yonelinas[8]. Encoding tasks that emphasize more active engagement with the stimuli may enhance performance. While our encoding task—reading a sentence aloud—encouraged participants to semantically engage with the words, it did not require them to generate content or engage in deep elaboration other than rating the degree of knowledge they have of the presented words. Accordingly, our encoding instructions may be more aligned with those employed by Toth & Parks [7] and Parks, Jacoby, & Yonelinas [8], which also showed age-related decline in automatic retrieval. Thus, while speculative, it is possible that part of our observed age-related decline in automatic memory reactivation could be related to encoding differences. Future work manipulating the encoding strategies could directly test this possibility.

**S5 Table. Sample Past Studies of PDP Automatic Estimates and Aging**

| Study | Sample Size (Age) | Encoding Task | Direction of Effect | Statistics |
| --- | --- | --- | --- | --- |
| Jennings, J. M., & Jacoby, L. L. (1993). Automatic Versus Intentional Uses of Memory: Aging, Attention, and Control. *Psychology and Aging*, *8*(2), 283–293. https://doi.org/10.1037/0882-7974.8.2.283 | 24 YA (*M=*19.5, SD=N/A) and 24 OA (*M*=73.8, SD=N/A) | Solved anagrams of each word and then read the aloud | YA = OA | p=0.82* |
| Rybash, J. M., Santoro, K. E., & Hoyer, W. J. (1998). Adult Age Differences in Conscious and Unconscious Influences on Memory for Novel Associations. *AGING NEUROPSYCHOLOGY AND COGNITION*, *5*(1), 14–26. https://doi.org/10.1076/anec.5.1.14.26 | 48 YA (*M=*19.3, SD=N/A) and 48 OA (*M=*73.6, SD=N/A) | Generated and wrote a meaningful sentence | YA = OA | N/A |
| Toth, J.P., Parks, C.M. (2006) Effects of age on estimated familiarity in the process dissociation procedure: The role of noncriterial recollection.  *Memory & Cognition* 34, 527–537. https://doi.org/10.3758/BF03193576 | 36 YA (*M=*19.8, SD=1.8) and 36 OA (*M=*71.1, SD=4.4) per condition | Read each word aloud | YA > OA | p<0.001 |
| Parks CM, DeCarli C, Jacoby LL, Yonelinas AP. (2010). Aging effects on recollection and familiarity: the role of white matter hyperintensities. Neuropsychol Dev Cogn B Aging Neuropsychol Cogn. 17(4):422-38. doi: 10.1080/13825580903469838. | 20 YA (*M=*18.9, SD=0.85) and 17 OA (*M=*72.4, SD=4.6) | Shallow (does the word contain “O” or “U”) and deep encoding (is this word pleasant or unpleasant) | YA > OA | p<0.001 |

***** value estimated from reported statistics

**Supplementary Note 6**

**Covariance of predictors**

To assess potential multicollinearity among predictors, we examined the pairwise correlations between the predictor variables used in each model.

VDT: The dependent variable (accuracy) was excluded from this analysis, as covariance or correlation is only meaningful between predictors (Age, standardized Shipley, Mean Similarity, Mean Word Length, Mean Concreteness, Mean Frequency). All absolute pairwise correlations were below 0.3 (range: -0.12 to 0.2), indicating low linear relationships between predictors and suggesting that multicollinearity is unlikely to be a concern in this analysis. Therefore, no additional variable reduction or adjustment was deemed necessary.

VCT: We did not compute the covariance between Age and Automatic estimates because Age is a categorical variable with two levels. Covariance is defined only between two continuous, numeric variables. Since Age is a factor, it does not have a meaningful variance or covariance in the conventional sense. Instead, differences in Automatic estimates across age groups were appropriately assessed using a t-test.

**References**

1. Baguley T. Serious stats: A guide to advanced statistics for the behavioral sciences. 2012.

2. Meadows Research. https://meadows-research.com/. 2023.

3. Majewska O, McCarthy D, van den Bosch JJF, Kriegeskorte N, Vulić I, Korhonen A. Semantic Data Set Construction from Human Clustering and Spatial Arrangement. Computational Linguistics 2021 March 1;47(1):69–116.

4. Merriam Webster. https://www.merriam-webster.com/wordfinder. 2024.

5. Jennings JM, Jacoby LL. Automatic versus intentional uses of memory: Aging, attention, and control. Psychology and Aging 1993;8(2):283–293.

6. Rybash JM, Santoro KE, Hoyer WJ. Adult Age Differences in Conscious and Unconscious Influences on Memory for Novel Associations. Aging, Neuropsychology, and Cognition 1998;5(1):14–26.

7. Toth JP, Parks CM. Effects of age on estimated familiarity in the process dissociation procedure: The role of noncriterial recollection. Memory \& Cognition 2006;34(3):527–537.

8. Parks CM, DeCarli C, Jacoby LL, Yonelinas AP. Aging effects on recollection and familiarity: the role of white matter hyperintensities. Neuropsychology, Development, and Cognition. Aging, Neuropsychology and Cognition 2010;17(4):422–438.
