# Supplementary figures and images for: Aging and episodic memory specificity: Evidence challenging a domain-general pattern separation decline
Source: PLoS One. 2025 Nov 24;20(11):e0336045. doi: 10.1371/journal.pone.0336045 (PMC12643304; doi:10.1371/journal.pone.0336045)

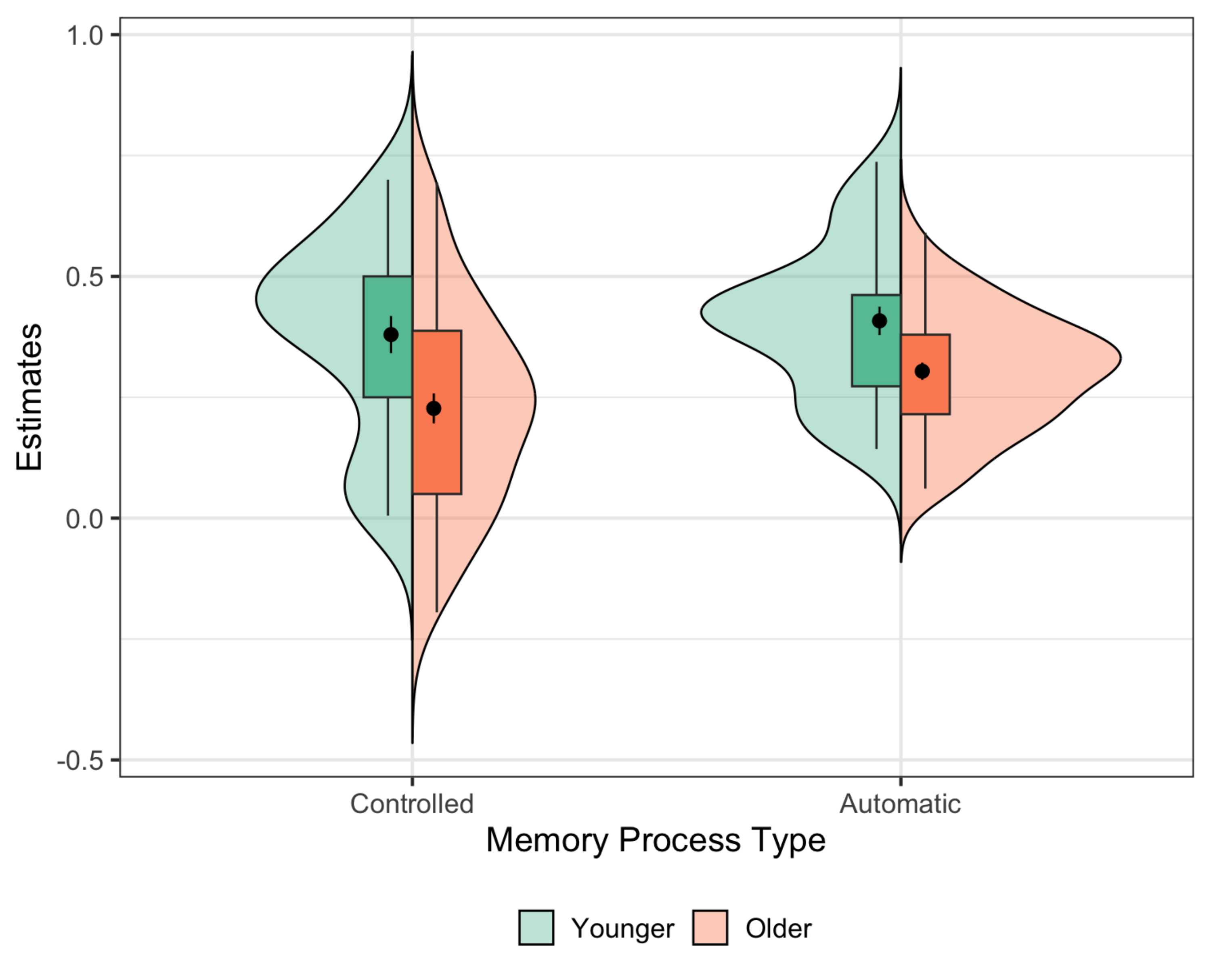

Supplement: S1 Fig — Within the sample of individuals who reported English as their first language, effects in the VCT remained the same as the full sample. Older adults had reduced controlled and automatic estimates compared to younger adults. (TIFF) [file pone.0336045.s002.tiff]

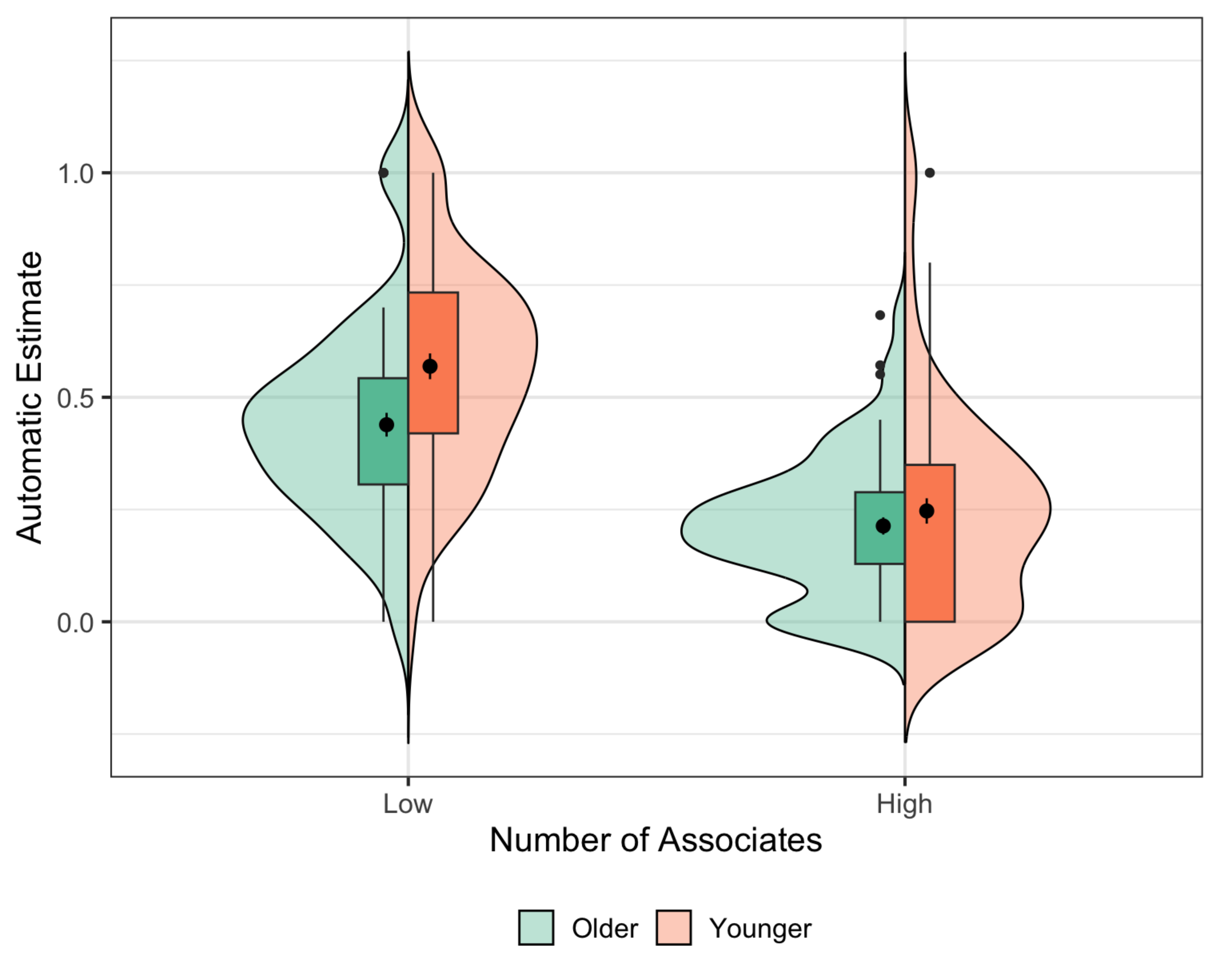

Supplement: S2 Fig — Individuals who studied words with a greater number of common stem-completion associates had more difficulty automatically reactivating the precise studied word at test (p < 0.0001), and this effect did not significantly differ across age groups. (TIFF) [file pone.0336045.s003.tiff]

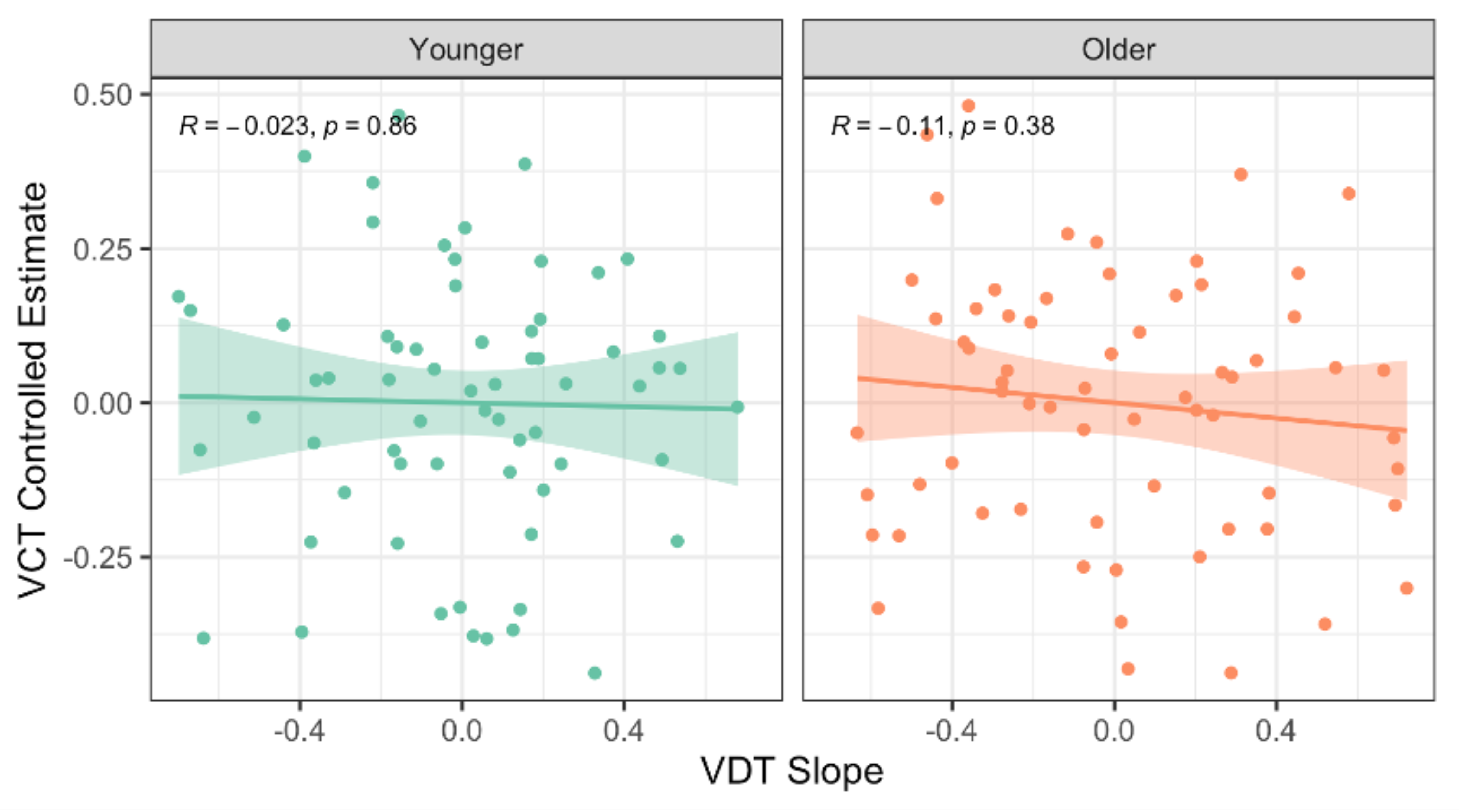

Supplement: S3 Fig — VCT = Verbal Completion Task; VDT = Verbal Discrimination Task. There were no significant correlations between controlled estimates from the VCT and similarity slopes from the VDT in either younger or older adults. (TIFF) [file pone.0336045.s004.tiff]

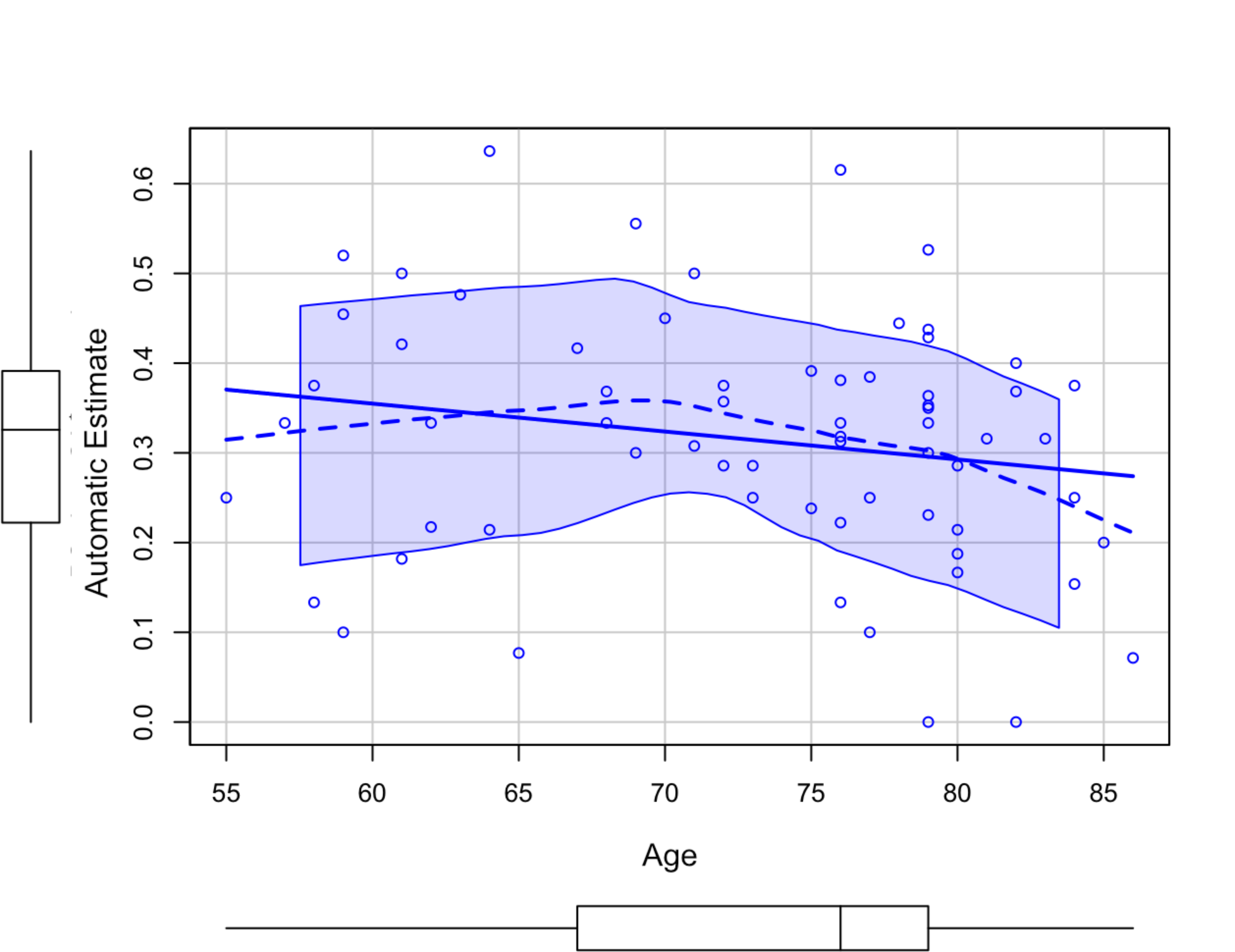

Supplement: S4 Fig — VCT = Verbal Completion Task. There were no significant correlations between automatic estimates from the VCT and age with the full older adult sample. (TIFF) [file pone.0336045.s005.tiff]
