# Supplementary material for: Aging and episodic memory specificity: Evidence challenging a domain-general pattern separation decline
Source: PLoS One. 2025 Nov 24;20(11):e0336045. doi: 10.1371/journal.pone.0336045 (PMC12643304; doi:10.1371/journal.pone.0336045)
Supplement: Attachment — Submitted filename: Response to Reviewers.docx [file pone.0336045.s006.docx]

**Response to Reviewers**

**Author Note:** Dear Reviewers, we appreciate the time and effort you have devoted to evaluating our manuscript. We are grateful for the thoughtful comments and constructive suggestions, which have significantly contributed to improving the clarity and quality of our work. Please find attached a detailed, point-by-point response to each comment, with our responses provided in blue. All page and line references refer to the revised manuscript with tracked changes. As the tracked changes formatting differs across Word (.doc) files and PDF, we have provided both the Word doc page and line number as well as the PDF page and line number for your reference.

**Reviewer 1**

**Summary**: The authors did a good job of incorporating prior research, theory, and methodology into their approach. The manuscript was well written, and the figures worked well to display the findings. The authors examined whether pattern completion and/or pattern separation are impaired in older adults versus younger adults using several complimentary tasks: the Mnemonic Similarity Task (visual), and two novel tasks: Verbal Discrimination Task (verbal) and the Verbal Completion Task (verbal). As expected, based on prior similar studies using the MST, they found that older adults exhibited impaired “Old”-Lure proportions, Target vs Lure d’, and Lure vs Foil d’, but intact “Old”-Target proportions, “Old”-Foil proportions, and Target vs Foil d’. Importantly, there was a significant interaction between age group and d’ type (Target-Lure d’ vs. Lure-Foil d’). This indicated that the older adults were more likely to treat lures like targets (and less like foils) than the younger adults. For the VDT, they found impaired “Old”-Lure proportions, “Old”-Foil proportions, Target vs Foil d’, and Target vs Lure d’, but intact “Old”-Target proportion and Lure vs Foil d’. Importantly, there was no impairment in Lure vs Foil d’, suggesting that memory specificity was intact in the semantic domain. However, neither younger nor older adults were able to discriminate lures from foils in the VDT (floor effect), making it difficult to determine whether there was an age-related impairment in memory specificity. Direct comparisons across groups between the Lure vs Foil d’ for the MST and VDT revealed more age-related differences for the MST than the VDT. Follow-up analyses of the effect of language familiarity or semantic-similarity on performance did not appear to be relevant the group comparisons of memory specificity in the semantic domain. For VCT, they found impaired Inclusion proportion as well as impaired estimates of automatic and controlled retrieval, whereas the Exclusion proportion was intact. There was no significant correlation identified between the VDT slope (thought to reflect pattern separation) and the automatic estimate from the VCT (thought to reflect pattern completion).

**Author response:** Thank you for your thoughtful and constructive comments. We appreciate your recognition of our integration of prior research, theory, and methodology, as well as your positive remarks about the writing and the clarity of the figures. We are happy to hear that the overall structure and presentation of the study were effective in conveying the key findings. Your feedback directly contributed to strengthening both the clarity and precision of the manuscript.

**Concerns**:

1. The authors found that older adults exhibited different levels of memory specificity on the MST than the VDT (i.e., lure vs foil d’) and stated “This suggests that the effect of age on performance depends on the task type, where greater declines emerge on the Object MST”. This conclusion is difficult to support given the floor effects for the Foil vs Lure d’ for both groups on the VDT. If both groups were above chance on the VDT and the same significant interaction emerged, this would be strong evidence for their claim. It appears they have created a task in which it is impossible for humans to discriminate between lures and foils, which is the critical type of discrimination needed to justify the claim they are trying to make about memory specificity being differentially impaired across tasks in older adults, i.e., that memory specificity is not a domain-general type of impairment that occurs during aging.

Author response: Thank you for raising this thoughtful and important comment. Specifically, here you raise the concern that the different effects of age on memory discrimination across modalities could reflect a floor effect since we failed to solicit significant foil-lure discrimination in our VDT design.

We agree that our primary conclusions depend on a robust impact of similarity on memory in both the MST and VDT. Indeed, this is why we cautioned the reader that, “false alarm rates to foils were higher in the VDT (27% for younger adults, 39% for older adults) than in the MST (3% for younger adults, 3% for older adults), suggesting that the novel foils may have been too similar to the studied items in the design” (**Word doc** page 21 lines 578-581; **PDF** page 23 lines 481-484). In our original design, we did not consider the degree of similarity of each stimuli (including the foils) to the rest of the stimuli. Thus, we revised the manuscript to state that any conclusions using *d’* “would be premature, however, because behavioral performance suggested that our semantic similarity manipulation was less effective than the object similarity in the MST; specifically, false alarm rates to semantic foils were higher than those in the MST and closer to lure false alarms in the VDT, reducing our ability to measure the impact of semantic relatedness on false alarm rates” (**Word doc** page 21 lines 592-596; **PDF** page 24 lines 496-501).

To overcome this hurdle, we completed a comprehensive series of semantic similarity analyses which compared the similarity of each new word, regardless of our initial lure vs. foil designation, to the full set of studied words. This regression analysis revealed a robust relation between semantic similarity and false alarm rates (β=-0.13, SE=0.03, p<.0001). We used the slope of this relationship in each participant as an alternative measurement of how semantic similarity influenced memory discriminability. So, by shifting our analytic approach, we achieved your recommendation that both groups show above chance discrimination on the VDT. Critically, it’s this statically robust slope that we entered the cited interaction analysis–not the insignificant lure vs. foil d’.

We hope our explanation clarifies that we agree with your reasoning and that we prioritized identifying a robust influence of semantic similarity on memory before moving forward with modality comparisons.

We also carefully re-read our results and discussion sections to identify aspects of our writing that may have led to the confusion over which metric was used in the critical interaction analysis. To make it clearer, we added a clarification on **Word doc** page 23 in lines 674-675; **PDF** page 27 lines 558-560 saying: “To this end, we were interested in directly comparing age-related difference in mnemonic discrimination across the Object MST (d’a for lure vs. foil trials) and the VDT (slope)”.

2. For the results supporting the analyses in Figure 5, the authors note there was no significant relationship between independent estimates of pattern completion from the VCT and pattern separation from the VDT. Yet, it is possible that these two estimates are uncorrelated because they are from two different tasks. It would be stronger evidence for their claim if they could show that independent estimates of pattern separation from both tasks were significantly correlated and/or that independent estimates of pattern completion from both tasks were significantly correlated. Against that backdrop, their finding that pattern separation and pattern completion were uncorrelated would be more convincing.

**Author response:** We were not exactly clear what was being asked here, but we understood your suggestion to be that stronger evidence for the lack of correlation between pattern separation (PS) and pattern completion (PC) would come from demonstrating converging estimates of each construct across tasks – for example, obtaining independent estimates of PS from both the VDT and VCT (or PC from both), and then correlating those. We interpreted your logic to be that if same-construct estimates converge across tasks, this would bolster confidence in our interpretation that PS and PC are separable processes, especially if, against that backdrop, the lack of correlation between PS and PC holds.

We agree that this would be a strong approach if the tasks in question provided independent estimates of the same construct. However, in our design, the VCT was explicitly intended to measure PC, and the VDT was designed to measure PS. While it is technically possible to derive a PS-like estimate from the VCT (e.g., by inverting the PC estimate), or a PC-like estimate from the VDT, such measures would not be independent. They would be inherently mathematically coupled. For example, the PS estimate from the VCT would simply be the inverse of the PC estimate, meaning any resulting correlation (or lack thereof) would be uninterpretable. This artificial dependency would violate the assumptions needed to meaningfully assess whether PS and PC are dissociable.

Thus, while we agree in principle that cross-task convergent validity would strengthen the argument, it would require a different experimental design in which multiple tasks provide independent estimates of each construct. This is an excellent direction for future work, and we now acknowledge this more explicitly in the revised manuscript: “another important consideration is our reliance on single tasks to estimate pattern separation and pattern completion. Ideally, convergent validity across multiple independent tasks measuring the same constructs would strengthen confidence in these interpretations. Achieving this would require an experimental design that systematically includes multiple complementary tasks to provide convergent evidence for each cognitive process. While this was beyond the scope of the present study, we acknowledge this as an important direction for future research to enhance construct validity and better characterize the mechanisms underlying age-related memory changes” (**Word doc** page 32 lines 1006-1023; **PDF** page 39 lines 833-840).

**Minor Concerns:**

1. Abstract: Words like “change” and “decline” are used, but given the non-longitudinal, group-based comparisons these types of words are not accurate. The results should be described in terms of deficits or impairment.

**Author response:** Thank you for pointing this out. We have replaced them with “deficits” and “differences” instead, as seen on **Word doc** page 2 lines 45- 46; **PDF** page 2 lines 46-48.

2. Page 17, line 343: “confirming desirable features of the MST design in our sample”. What desirable features are the authors referring to?

**Author response:** We clarified this with: “This pattern was consistent with the MST’s intended design in which lures elicit higher false-alarm rates than foils, reflecting their perceptual similarity to targets”, on **Word doc** page 19 lines 531-533; **PDF** page 22 lines 443-445.

3. Page 19, line 389: “This conclusion would be premature however, because our manipulation of semantic similarity was not as pronounced as the object similarity manipulation in the MST”. Please make it clearer how the authors are making the determination that semantic similarity was not as pronounced as on the object similarity in the MST. It is not clear what characteristics or findings they are looking at to make that decision.

**Author response:** Thank you for pointing this out. We have changed it to: “This conclusion would be premature, however, because behavioral performance suggested that our semantic similarity manipulation was less effective than the object similarity in the MST; specifically, false alarm rates to semantic foils were higher than those in the MST and closer to lure false alarms in the VDT, reducing our ability to measure the impact of semantic relatedness on false alarm rates.” on **Word doc** page 21 lines 592-596; **PDF** page 24 lines 496-501.

4. Page 29, line 610. The authors state that a limitation of their behavioral study was that it did not include neuroimaging data. Reporting on behavior alone within a conceptual framework, but without accompanying neuroimaging data, is not a limitation.

**Author response:** Thank you for this suggestion. We agree, and we have reframed the section as a future direction: “In future studies, neuroimaging data could further connect these behavioral findings to neural representations in the hippocampus and neocortex. Our focus on refining behavioral assessments offers a useful complement to prior neuroimaging work, and we hope it inspires future research investigating how the aging brain encodes and automatically retrieves semantic details.”( **Word doc** page 33 lines 1024-1028; **PDF** page 39 lines 841-845).

**Reviewer 2**

**Summary**: The present article, "Aging and episodic memory specificity: Evidence challenging a domain-general pattern separation decline" explores (1) the loss of specificity in healthy aging, found in visual episodic memory, for conceptual similarity and (2) how pattern separation and pattern completion are interdependent for this situation. The study is based on three experimental tasks, a classic mnemonic similarity task, an adapted version manipulating semantic similarity and a verbal completion task to assess semantic pattern completion.

The topic is timely and relevant. Increasing data support the loss of specificity for visual episodic memory. Yet, little is known for conceptual material. In the same vein, it is supposed that pattern completion is interdependent with pattern separation, but behavioral results remain mixed.

Overall, the article is well written, based on an original and adapted methodology, and proposes an interesting contribution to the topic. The introduction offers a comprehensive review of the literature. The experimental paradigms are well designed. The statistical analyses are correctly done and the conclusion is consistent with the data.

Nonetheless, I will have relatively major comments (see below) that should be addressed leading to a major revision.

**Author response:** Thank you for your thoughtful review. We sincerely appreciate your recognition of the originality of our methodological approach and the clarity of the manuscript. We are especially grateful for your remarks on the rigor of the experimental design and statistical analyses, as well as your acknowledgment that this work adds meaningfully to a complex and evolving literature. Your insights were both encouraging and helpful as we revised the manuscript.

**## Major Comments**

One of the study’s aims, stated in the introduction section, is “3) We accounted for other cognitive processes, such as executive functioning or visual-spatial processing”, but no mention of the objective is made in the abstract. I suggest adding this information there.

**Author response:** Thank you for your suggestion. We have modified the abstract to include these details: “The current study aimed to investigate whether aging affects memory discrimination for semantically similar content, using tasks that minimize reliance on visual-spatial processing and executive functioning, both of which tend to decline with age.” on **Word doc** page 2 lines 34-37; **PDF** page 2 lines 34-37.

I am also confused about the control mentioned. The authors used a PDP (process dissociation procedure) procedure, which is suitable to account for the attentional and controlled process, but it is unclear how the visual-spatial processing is taken into account. No further mention of visuo-spatial processing is done in the article. This mention should be removed or relevant data should be added.

**Author response:** Thank you for pointing this out. We acknowledge that our original phrasing “accounting for” visual-spatial processing may have been misleading. To clarify, we employed verbal tasks that do not rely on visuo-spatial processing. We have revised the manuscript accordingly to make this clearer: “We accounted for executive functioning and used tasks that were less reliant on visual-spatial processing, since these also show age-related decline and contribute to task performance.” on **Word doc** page 6 lines 132-134; **PDF** page 6 lines 132-134.

Moreover, it is stated that “we administered a neuropsychological test battery to older adults only”, but that these data will not be discussed here. Nonetheless, executive and visuo-spatial results from the neuropsychological assessment should be used to account for the third objective of the paper. By the way, please add, at least as supplementary data, the list of the tests used.

**Author response:** The changes we have made above will make this section clearer; we did not use any data from the neuropsychological tests in this study because all of the results in this manuscript focused on age group comparisons, and we did not collect any neuropsychological data for the younger adults. We had only collected the neuropsychological data from older adults to inform additional MRI analyses to answer other questions about aging, which we will present in a separate manuscript. However, we have included a list of tests we used in the Supplementary Method on **Word doc** page 4.

On a statistical level, the age range is very large for the older adults considering that aging decline accelerated around the 70’s. The analysis method should control for it. In the same idea, the education level of the older adults is very high and significantly different from the one in the young adults group. This variable should also be controlled.

**Author response:** Thank you for this insightful comment. To ensure that variability in chronological age within each group did not confound our findings, we conducted supplementary analyses incorporating age as a continuous covariate centered within each group (i.e., within-group age). Across all models and tasks, the inclusion of within-group age did not significantly influence the results, indicating that the observed group differences were not driven by age variability within the age groups. These results are now reported in the Supplementary Materials (see Supplementary Note 5 on page 10], and the main findings remained consistent, suggesting that our effects are not solely driven by the oldest individuals in the sample.

With respect to education, we respectfully disagree with the suggestion to include it as a covariate in our analyses. While education is commonly used as a proxy for cognitive reserve or premorbid cognitive ability in older adults, it is less appropriate in a younger sample, especially one currently enrolled in post-secondary education. Educational attainment is still ongoing for most of our young adult sample, and thus is not directly comparable across age groups.

Instead, following neuropsychological conventions, we relied on a standardized measure of crystallized knowledge to estimate baseline cognitive ability: all participants completed the Shipley Vocabulary test (a measure of vocabulary), and standard scores were computed relative to age-matched normative data. These scores provide a more robust estimate of premorbid functioning than education level alone (Lezak et al., 2012).

Importantly, there was no significant difference in standardized Shipley scores between younger and older adults, suggesting comparable levels of premorbid cognitive ability across groups. This supports our decision not to statistically control for education. We also considered the potential impact of English-as-a-first-language status on performance and included follow-up analyses to examine this issue (see Supplementary Note 1 on page 4).

Citation:

Lezak, M. D., Howieson, D. B., Bigler, E. D., & Tranel, D. (2012). *Neuropsychological assessment* (5th ed.). Oxford University Press.

I would recommend exploring the correlation matrix between all variables and/or to use, at least ANCOVA, or better, hierarchical regressions or generalized linear mixed as used after (by the way, a yes/no recognition task is more like a Bernoulli distribution than a normal distribution which shouldn’t be processed by ANOVA, see Dixon, 2008).

**Author response:**

We appreciate the reviewer’s comment regarding the appropriate statistical treatment of yes/no recognition data. To clarify, our analyses already included a generalized linear mixed-effects model (GLMM) with a binomial link function using glmer() to model trial-level accuracy (correct/incorrect) as a Bernoulli outcome for the VDT. This model incorporated both fixed effects (e.g., similarity, age, cognitive measures) and subject-level random effects, as reported in the manuscript (see VDT Results). To make this clearer, we added: “We then entered each into its own generalized linear mixed model via the lme4 package (v1.1.35.1) to assess how it predicted trial-level foil/lure accuracy (coded as correct=1, incorrect=0) (see Fig 2C). These models used a binomial distribution with a logit link, appropriate for binary outcomes.” On **Word doc** page 22 lines 647-651; **PDF** page 26 lines 537-541. Our model specification can also be found in our Supplementary Methods, page 1.

As for the MST, we analyzed d′ scores, which are continuous, normally distributed summary measures of discriminability. Importantly, we only had one d′ score per participant per discriminability comparison. These scores were appropriately analyzed using ANOVA, as is standard (see Stark et al., 2015).

We assessed the pairwise relationships among the predictor variables used in our models to check for potential multicollinearity. Covariance and correlation matrices were computed on the predictor subset, excluding the dependent variable.

The results indicated that all absolute pairwise correlations were below 0.3 (range approximately from -0.28 to 0.14), showing low linear associations among predictors. The variances of the standardized predictors were close to 1, as expected.

These findings suggest that multicollinearity is unlikely to have affected our regression analyses, and no further variable reduction or adjustment was necessary. Please see Supplementary Note 6 for more details.

Some statistical details are also missing such as the use of bilateral or unilateral p-values tests, the control of outliers for accuracy and reaction times, other assumptions checks (normality), and so on.

**Author response:** Thank you for pointing this out. We have revised the manuscript to clarify our statistical approach. Specifically, we now note in table footnotes that all p-values are two-tailed. Additionally, we have included a section titled “Statistical Approach” at the end of the Methods section, which now reads:

“All statistical tests were two-tailed with a significance threshold of α = 0.05. For parametric tests (e.g., t-tests, linear mixed models, ANOVAs), assumptions of normality were tested using Shapiro-Wilk tests. Where post hoc comparisons were conducted, we applied Sidak corrections for multiple comparisons. To reduce the influence of extreme outliers, we applied the winsorize() function in R (69), which replaces values below the 5th percentile with the 5th percentile value, and values above the 95th percentile with the 95th percentile value. For binary outcome data (e.g., accuracy in the VDT), we used generalized linear mixed-effects models with a binomial distribution. Model fit was assessed using AIC, and continuous predictors were scaled prior to analysis.”

Please comment on the possible bias related to intentional memorizing content in the semantic adaptation of the MST vs. the incidental learning done in the standard MST.

**Author response:** Thank you for catching this – this was actually a mistake in the manuscript made on our part because we used intentional encoding for the MST. We modified this on **Word doc** page 15 lines 445-445; **PDF** page 18 lines 376-380: “The MST included an encoding phase in which participants were instructed to remember objects for a later memory test.”

Perceptual information is richer than conceptual ones. As analogous signals, perceptual information are filled with details (e.g. color nuances, resolution, context, points of view, and so on) that are not presented in a verbal concept (there is a multiple of possible representations of an ELEMENT). Consistent with this idea, phonological similarity induces a similar loss of specificity. Consequently, it is difficult to conclude that the interaction between age and the nature of the stimuli is supported by a preserved processing of the conceptual information. One could imagine that the effect is mainly driven by the degree of details convey in the stimuli used (see also the sentence l. 390 “semantic similarity was not as pronounced as the object similarity manipulation in the MST”). Could the authors better justify their conclusions or acknowledge this hypothesis as a limit of their study. Otherwise, please add more nuanced to the discussion (e.g. “our results add to emerging evidence that age-related decline in memory specificity is restricted to perceptual domains...” ).

**Author response:** This is very insightful, thank you. We understand your concern that the interaction between age and stimulus type may be influenced not solely by the nature of conceptual vs. perceptual processing, but by differences in the representational complexity or richness of the stimuli themselves. As you noted, perceptual information often contains more fine-grained, detail-heavy features (e.g., visual resolution, spatial perspective), which may place greater demands on memory fidelity compared to more abstract or unitary conceptual information. This could help explain why age-related declines appear more pronounced in the perceptual domain.

We agree this is an important alternative interpretation and have revised our discussion to include it. On **Word doc** pages 28-29, lines 818-850; **PDF** page 33 lines 697-707, we now state: “this apparent preservation in semantic discrimination with age may also be related to inherent differences in the representational complexity across domains. Perceptual stimuli often contain more nuanced, detail-rich features (e.g., color, spatial resolution, viewer perspective) than conceptual stimuli, and this complexity may place a greater burden on memory fidelity, making it more susceptible to age-related decline. Therefore, the observed interaction between age and stimulus type may reflect these differences in complexity, rather than the comparative preservation of conceptual details in memory. Indeed, alternative accounts of age-related memory decline, such as the 'cluttered memory' hypothesis (85), suggest that older adults may retain more irrelevant features, which can interfere with tasks requiring high representational fidelity. From this perspective, semantic representations, having fewer details, may be less susceptible to such interference.”

We appreciate your insight and the opportunity to integrate this important nuance into our interpretation.

**## Minor Comments**

- All the data should be uploaded on the OSF as dropbox is a suitable and durable sharing method (the data remain on the folders of someone specifically and the access could be easily removed).

**Author response:** We have now uploaded the data under the “Files” tab: https://osf.io/jkcg9/?view_only=95d52ace925a4e6ab339d760e50ad351.

- The pictures should be uploaded with higher quality (e.g. 600 DPI).

**Author response:** Thank you for pointing this out; we have edited all figures to have a higher resolution and re-uploaded them. These changes will be reflected when downloading each figure.

- Update the abstract to mention the aim to add the classical MST task for a subset of participants (i.e. direct comparison of visual vs semantic similarity, see l).

**Author response:** Thank you for your suggestion. We have updated the abstract on **Word doc** page 2 lines 39-41; **PDF** page 2 lines 39-41: “Sixty-six younger adults and 66 older adults completed these tasks, and a subset also completed the Object MST (younger n=33 and older n=35) to allow for a direct comparison between visual and semantic similarity.”

- For the same reason, update the introduction section presenting the aims of the study (l-139) to explicitly present the rational to “We additionally included the standard object MST for comparison with the VDT for a subset of participants”. If it is an objective of the study, it should be presented with the other ones (l. 125-131) and if it a secondary or exploratory aims, it should be mentioned as it (see l. 210 “Midway through data collection, we became interested in comparing performance in the VDT with the visual object MST.”).

**Author response:** Thank you for your suggestion – as it was not an initial aim of ours, we left it as it is. However, taking in your suggestion we have clarified that this was an exploratory aim, on **Word doc** page 7 line 142; **PDF** page 7 line 142.

- Please add more details about the power analysis. Was it computed for a simple t-test/ANOVA test or did it account for the hierarchical levels or possible covariables.

**Author response:** Thank you for pointing this out. We added more details: “We predetermined this sample size from an a priori power analysis to obtain 80% power. The analysis was based on a two-tailed independent samples t-test comparing younger and older adults on the pattern separation index using an effect size of d=0.59 derived from a previous study (15)” on **Word doc** page 8 lines 176-179; **PDF** page 8 lines 173-176.

We chose to base our power analysis on a simple between-group comparison for two reasons. First, at the time of study planning, this comparison reflected the primary research question motivating our sample size – namely, whether age differences would emerge in pattern separation performance. Second, although we used hierarchical modeling in our final analyses to account for trial-level variance, power calculations for such models are highly dependent on assumptions about trial structure, random effects, and covariance parameters that can be difficult to estimate reliably in advance. We therefore opted for a more conservative and transparent approach based on established group-level effects from prior research.

- Please provide more details on the ethical approval (e.g. approval id or number).

**Author response:** We added all the necessary details as mandated by PLoS ONE in our Methods section, on **Word doc** page 9 lines 200-202; **PDF** page 9 lines 197-199 saying: “All participants gave their informed written consent before the administration of the tasks, and all methods were approved by the University of Toronto Social Sciences, Humanities, and Education research ethics board.”

- In general, please add Notes to the tables to provide details on the acronyms and abbreviations (SD, F, M, MST...). Please also add where the group comparisons were significant.

**Author response:** Thank you for pointing this out. We added relevant footnotes to both Tables 1 and 2. The bolded font p-values in the right column indicate significance.

- I would not agree with the following statement (l. 206-208) “this way, it is more likely that any observed age-related memory impairments are driven by mnemonic changes rather than visual acuity or perceptual changes.”. The pattern separation literature is heavily based on representational-hierarchical approach which did not consider a difference in nature between memory and perceptual processes (see Kent et al., 2016; see also embodied view of cognition Mille et al., 2021).

**Author response:** Thank you for this theoretical note. We adapted this section in the manuscript with your suggested changes on **Word doc** page 10 lines 217-221; **PDF** page 10 lines 214-218 “Thus, this approach minimizes the influence of low-level visual differences (e.g., visual acuity) across age groups, allowing us to better isolate age-related changes in representational fidelity. While memory and perception are closely intertwined processes (49,50), this design reduces the likelihood that performance differences are solely attributable to low level perceptual factors.”

- It is odd to mix up separated at a similar level the verbal stimuli and the tasks using them. I suggest using a hierarchical structure with something like “Semantic tasks” at a higher level and within it “Verbal stimuli”, “Intentional Encoding Task”.

**Author response:** Thank you for your suggestion; we changed it to a hierarchical structure so that the Verbal Completion Task and the Verbal Discrimination Task were on the same heading levels, and within each were subheadings of: Material, Encoding phase, and Retrieval phase. These changes are reflected starting from **Word doc** page 11; **PDF** page 11.

- L. 321-325 : the paragraph is mainly a redundant part of the method, please remove it.

**Author response:** Thank you for pointing this out. We removed this paragraph, as reflected on **Word doc** page 14; **PDF** page 16.

- Please provide the exact formula used to compute the discriminability index (l. 329).

**Author response:** Thank you; we updated our Results to include the formula on **Word doc** page 19 line 518; **PDF** page 21 line 428.

- Letters used in mathematical expressions should be in italic (F, t, p...) like in F(1,132) = 333.34, p < .

**Author response:** Thank you for pointing this out. We have made the requested formatting changes throughout the manuscript.

- Please change the verb “tended” (l. 386) as it could imply that the effect is not really significant.

**Author response:** Thank you for pointing this out; this sentence has now been replaced on **Word doc** page 21 lines 588-589; **PDF** page 24 lines 492-493 with: “indicating that older adults were more likely to falsely identify lure items as studied targets”

- Please remove this statement for the results “semantic similarity was not as pronounced as the object similarity manipulation in the MST.” or provide more explanations about it, here.

**Author response:** Thank you for pointing this out; this sentence has now been replaced with: “This conclusion would be premature, however, because behavioral performance suggested that our semantic similarity manipulation was less effective than the object similarity in the MST; specifically, false alarm rates to semantic foils were higher than those in the MST and closer to lure false alarms in the VDT, reducing our ability to measure the impact of semantic relatedness on false alarm rates.” on **Word doc** page 21 lines 592-596; **PDF** page 24 lines 496-501.

- Please use consistent terminology between semantic and domain-general terms (e.g. l. 522).

**Author response:** Thank you for your suggestion; we replaced it with: “First, we tested whether older adults showed a domain-general decrease in memory specificity across semantic and perceptual domains” on **Word doc** page 27 lines 792-793; **PDF** page 31 lines 561-563. We hope this addresses your concern.

- What the authors mean by "reflect a lack of interest in perceptual details” (l. 539-540)?

**Author response:** Thank you for pointing this out. We clarified our intention on **Word doc** page 28 lines 811-817; **PDF** page 32 lines 692-696: “As older adults experience declines in detail-specific memory, they may also place greater value on meaningful, conceptually relevant information. This motivational shift may enhance semantic processing and neural specificity (hyperdifferentiation) for conceptual information (89–92), potentially benefitting performance in tasks like the VDT, which require fine-grained discrimination of conceptually similar words.”

- I would recommend being more nuanced about “a robust deficit in automatically recall memories” (l. 584) as numerous studies have reported the opposite and as the tasks constraints should be taken into account (e.g. trigram completion has been reported as being more demanding than a real automatic memory recall and to involve some executive components).

**Author response:** Thank you for pointing this out. We agree that terminology may imply generalization beyond what our results can support. Accordingly, we have changed “robust” to “marked” and “recall memories” to “reactivate previously studied items” on **Word doc** page 31 line 942; **PDF** page 37 line 801.

\## References

- Dixon, P. (2008). Models of accuracy in repeated-measures designs. *Journal of Memory and Language, 59*, 447–456.

- Kent, B. A., Hvoslef-Eide, M., Saksida, L. M., & Bussey, T. J. (2016). The representational–hierarchical view of pattern separation : Not just hippocampus, not just space, not just memory? _Neurobiology of Learning and Memory, 129, 99 106.

- Mille, J., Brambati, S. M., Izaute, M., & Vallet, G. T. (2021). Low-Resolution Neurocognitive Aging and Cognition : An Embodied Perspective. _Frontiers in Systems Neuroscience, 15, 687393.

**Author response:** Thank you for the helpful references! We incorporated the last two references into our manuscript.

**Reviewer 3**

**Summary**: This study investigates the impact of semantic relatedness on age differences in pattern separation and completion. The authors designed two new tasks, namely the verbal discrimination and completions tasks. A subset of participants also completed the standard version of the mnemonic similarity task (MST). The results showed that the age-related decline in memory specificity is stronger for perceptual than for semantic relatedness. Older adults were also impaired in pattern completion. The authors interpret these results as evidence against the view that typical aging is associated with a domain-general decline in pattern-separation abilities and a bias toward pattern-completion.

The study question is relevant and topical. Also, the effort the authors have made to design the new tasks, and to characterize the semantic relatedness of all list words, are remarkable. The results of this study will be informative for the cognitive aging scientific community. However, there are some limitations that need to be addressed, which I list below.

**Author response:** Thank you for your thoughtful and constructive review. We truly appreciate your recognition of the conceptual relevance of our study and the effort involved in designing the new tasks and characterizing semantic relatedness across our stimuli. Your comment that the results will be informative for the cognitive aging community is deeply encouraging, and we share your hope that this work contributes meaningfully to ongoing discussions around age-related changes in memory specificity. We’ve carefully considered the limitations you raised and have addressed them in the revised manuscript. Thank you again for your helpful insights.

1. The design of the present study includes some specific features that must be considered. First, the encoding procedure used for the VCT and VDT is likely to have oriented participants toward a deeper conceptual processing of the target words. Given that numerous previous studies have established that older adults benefit from semantic (and schematic) support, such an encoding strategy may have been especially beneficial to this group compared with younger adults, tempering age differences. Unless the authors disagree with this view, it should be acknowledged in the manuscript.

**Author response:**

Thank you for this thoughtful comment. You raise the important point that our sentence-reading encoding task may have encouraged deeper conceptual processing, which could potentially benefit older adults more than younger adults due to their reliance on semantic and schematic support. From this view, the encoding strategy may have tempered age differences and should be acknowledged.

We agree that semantic support during encoding can influence age-related memory performance, and that encoding strategies are an important consideration when interpreting these effects. Importantly, we intentionally selected the sentence-reading task to provide a modest level of semantic support that encourages all participants (both younger and older adults) to engage with the meaning of the target words. Our rationale was to reduce the likelihood that age differences would be driven by older adults’ difficulties with spontaneously adopting effective encoding strategies, which are known to vary with age (e.g. Craik & Rose, 2012). By offering this moderate but controlled support, we aimed to level the playing field regarding executive demands and strategy use, enabling us to more directly assess age differences in the quality of memory representations rather than confounding effects of strategic engagement.

We recognize that our original description may not have clearly conveyed this rationale. To address this, we have revised the Methods and Discussion sections to clarify the intention behind this encoding strategy and how it helps isolate representational memory differences by reducing confounding effects from executive or strategic factors.

Methods: “This sentence-based encoding task was deliberately chosen to provide a modest level of semantic support for all participants. Our aim was to reduce age-related variability in spontaneous strategy use – particularly older adults’ documented difficulties with self-initiated encoding – while minimizing executive demands” (see **Word doc** page 13 lines 379-381; **PDF** pages 14-15 lines 310-313).

Discussion: “Additionally, our choice of encoding task – sentence reading – could have influenced our findings. We intentionally designed it to support consistent encoding strategies across groups, but it is possible that the conceptual processing that it promoted could have disproportionately benefitted older adults, who tend to rely on semantic and schematic support.” (see **Word doc** page 32, lines 1002-1006; **PDF** page 38 lines 828-831)

Citation:

Craik, F. I., & Rose, N. S. (2012). Memory encoding and aging: a neurocognitive perspective. *Neuroscience and biobehavioral reviews*, 36(7), 1729–1739. https://doi.org/10.1016/j.neubiorev.2011.11.007

Second, encoding was explicit in the VCT and VDT but incident in the MST; could the authors explain this methodological choice, and how this is considered in the interpretation of the results?

**Author response:** Thank you for catching this – this was actually a mistake in the manuscript made on our part because we used intentional encoding for the MST. We modified this on **Word doc** page 15 lines 445-445; **PDF** page 18 lines 376-378: “The MST included an encoding phase in which participants were instructed to remember objects for a later memory test.”

Finally, from my understanding, there was no delay between encoding and test in the VCT whereas there was a 10-minute delay in the VDT – is that correct? If so, could the authors also explain this choice and its potential influence on the results?

**Author response:** Yes, there is a 10-minute delay just between VDT encoding and retrieval. The reason behind this choice is included within the manuscript: “There was also a 10-minute delay between VDT encoding and retrieval, in which participants were given a Sudoku puzzle. The purpose of this delay was to increase the difficulty of the task, as verbal memory recognition tests may be more prone to ceiling effects than cued recall tests, especially in younger adults [66].” on **Word doc** page 15 lines 428-432; **PDF** page 18 lines 376-377. We hope this addresses your concerns.

2. I believe that the authors could further discuss the implications of their results for the nature of memory representations in typical aging. For example, the concept of “gist” is insufficiently developed. The authors mention that older adults tend to rely more on gist memory (p. 3, lines 55-56), but do not define gists. Some relevant references could be cited in the introduction and discussion, such as Grilli & Sheldon (2022), and/or Greene & Naveh-Benjamin (2023), and/or Brainerd & Reyna (2015). Another hypothesis is that memory representations in healthy older adults are “cluttered”, that is, they include more irrelevant and no-longer relevant features, in addition to relevant features, which harms performance in memory tasks that require memory representations of high fidelity (Amer et al., 2023).

Grilli, M. D., & Sheldon, S. (2022). Autobiographical event memory and aging : Older adults get the gist. Trends in Cognitive Sciences, 26(12), 1079 1089. https://doi.org/10.1016/j.tics.2022.09.007

Greene, N. R., & Naveh-Benjamin, M. (2023). Adult age-related changes in the specificity of episodic memory representations : A review and theoretical framework. Psychology and Aging, 38(2), 67 86. https://doi.org/10.1037/pag0000724

Brainerd, C. J., & Reyna, V. F. (2015). Fuzzy-trace theory and lifespan cognitive development. Developmental Review, 38, 89 121. https://doi.org/10.1016/j.dr.2015.07.006

Amer, T., Wynn, J. S., & Hasher, L. (2022). Cluttered memory representations shape cognition in old age. Trends in Cognitive Sciences, 26(3), 255 267. https://doi.org/10.1016/j.tics.2021.12.002

**Author response:** This is very insightful, thank you. We have weaved it into our Discussion on **Word doc** page 29 lines 846-858; **PDF** page 33 lines 703-715: “Indeed, alternative accounts of age-related memory decline, such as the 'cluttered memory' hypothesis (85), suggest that older adults may retain more irrelevant features, which can interfere with tasks requiring high representational fidelity. From this perspective, semantic representations, having fewer details, may be less susceptible to such interference. Domain differences in complexity are also relevant for gist-based accounts of aging, in which older adults are argued to retain the general features of an experience, rather than its exact details (86–88). These types of representations may be more suited to semantic discrimination than more detail-sensitive perceptual discrimination. Thus, the dissociation we observe between perceptual and semantic memory decline may not reflect a fundamental domain-based distinction, but rather differences in the complexity of the stimuli themselves. This challenge of disentangling perceptual and conceptual contributions is a central issue for psychological research, and addressing it will inspire major methodological and theoretical innovations moving forward.”

3.A. The observation of a trend for age x similarity interaction effect on correct rejections in the VDT in the participants who reported English as first language is important. Figure 3 distinctly shows that in young adults there was no relation between semantic similarity and correct rejections, whereas these two variables are negatively associated in older adults. Considering that this analysis was conducted in a subsample, it is likely to be underpowered. Thus, if this effect existed, it would go against the authors’ conclusions, for example “our results add to emerging evidence that age-related decline in memory specificity is restricted to perceptual domains, compared to semantic ones” (p. 25, lines 535-537), or “In contrast to older adults’ mainly preserved memory specificity in the semantic domain” (p. 27, line 580). Unless the authors can argue against this, I believe that these interpretations should be nuanced.

**Author response:**

Thank you for highlighting the trend for an age by similarity interaction effect on correct rejections in the verbal discrimination task (VDT) within the English-first-language subsample. We understand your concern that, given the relatively small sample size, this effect may be underpowered and that interpreting this trend requires caution. Balancing the need to avoid overinterpreting statistical trends with recognizing the meaningful nuance this analysis offers was a challenge for us. We appreciate your comment as it prompted us to revisit this balance and acknowledge that our original language may have been overly cautious.

Accordingly, we have revised our interpretations to reflect this nuance: for example, we state “our results align with previous work suggesting that age-related decline in memory specificity may be more evident in perceptual domains than in semantic ones” but that “Our results should also be considered within the context of some limitations” on **Word doc** pages 28-32 lines 806-1023; **PDF** page 32-36 lines 685-818.

This revision also allows us to connect more explicitly with the findings of Ilyes et al., whose work similarly suggests that semantic memory specificity in aging may not be entirely spared, highlighting that this area warrants further investigation. We have incorporated this connection in the discussion to emphasize how our findings complement and extend the current literature.

We thank you for encouraging us to better articulate this complexity and hope this nuanced framing clarifies our position.

3.B. Related to this, the authors highlight the question of why older adults “do not show superior semantic discrimination” although they have “heightened semantic knowledge” (lines 561-562, p.26). It is important to keep in mind that the tasks designed in this study and those used in most similar studies (including that of Ilyés, Paulik, and Keresztes, 2024, SciRep), rely on episodic memory, although the conceptual similarity of targets and lures can be manipulated. Therefore, performance on these tasks is impacted by the fidelity of episodic memory representations, which declines with typical aging. This could partially explain why older adults’ performance on such tests is impaired although they have enhanced semantic knowledge. What do the authors think about this tentative interpretation?

**Author response:** Thank you for this insightful comment. We fully agree that while our task manipulates semantic similarity, performance is ultimately reliant on episodic memory processes, which are known to decline with age. This provides a compelling explanation for why older adults may not exhibit superior semantic discrimination in our task despite possessing richer semantic knowledge, as shown on **Word doc** page 28 lines 805-817; **PDF** page 32 lines 684-692.

4. Can the authors specify explicitly on what data the multiple linear regression model that aimed at comparing age differences between the object MST and VDT was conducted (p.21, lines 433-442)? My question aims to confirm that the dependent variable here corresponds to memory discrimination, since the authors report an age x task type interaction. If the dependent variable did not correspond to a mnemonic discrimination measure, then a three-way interaction (i.e., age x task type x stimulus, e.g., lure/foil) would be warranted to ensure that the effect can be interpreted as reflecting memory specificity rather than overall memory performance. Line 436 seems to indicate that, but making it explicit could be helpful for future readers.

**Author response:** Yes, we clarified this on **Word doc** page 23 lines 674-689; **PDF** page 27 lines 558-560: “To this end, we were interested in directly comparing age-related differences in mnemonic discrimination across the Object MST (d’a for lure vs. foil trials) and the VDT (slope) […] After scaling, these measures of memory discriminability served as the dependent variable in a multiple linear regression model, which examined how they were predicted by the interaction between age and task type.”

5. As highlighted by the authors p.27, the use of verbal materials and synonyms to manipulate semantic relations between stimuli is relevant but also comes with limitations. Mentioning ways to overcome such limitations in future studies could be helpful to readers. For example, some researchers have manipulated conceptual similarity for visual material (e.g., Frick et al, 2023; Naspi et al, 2021). Alternatively, the material used by Ilyés et al., 2024 also seems to overcome this limitation, as the related/unrelated lure manipulation comes from the rearrangement of word pairs (e.g., “police uniform” and “official uniform”).

Frick, A., Besson, G., Salmon, E., & Delhaye, E. (2023). Perirhinal cortex is associated with fine-grained discrimination of conceptually confusable objects in Alzheimer’s disease. Neurobiology of Aging, 130, 1 11. https://doi.org/10.1016/j.neurobiolaging.2023.06.003

Naspi, L., Hoffman, P., Devereux, B., Thejll-Madsen, T., Doumas, L. A. A., & Morcom, A. (2021). Multiple dimensions of semantic and perceptual similarity contribute to mnemonic discrimination for pictures. Journal of Experimental Psychology. Learning, Memory, and Cognition, 47(12), 1903 1923. https://doi.org/10.1037/xlm0001032

**Author response:** Thank you for raising this – we appreciate your suggestion and have added it in on **Word doc** page 31 lines 937-939; **PDF** page 36 lines 775-777: “Future researchers may also consider manipulating semantic relationships by altering conceptual similarity in visual stimuli or by rearranging word pairs with high and low similarity, as demonstrated by Ilyés et al.”

**Minor comments**

• I am not sure that the study by Delarazan et al. (2023, Learning and memory) cited p.4 (lines 92-94) can be described as investigating “perceptual and semantic” discrimination. Rather, the authors tested memory for narrative details, that is, “moments described” (i.e., how the story unfolded). While I acknowledge that this is close to the semantic domain, it must not be confused with manipulations of conceptual similarity such as the one in the present study or that of Ilyés et al., 2024.

**Author response:** Thank you for this important clarification. We understand that the Delarazan et al. (2023) study focused on memory for narrative details (i.e. how a story unfolds) which is related to but distinct from the type of conceptual or semantic similarity manipulations used in our study and that of Ilyés et al. (2024). To better reflect this distinction, we have revised our language to describe Delarazan et al.'s findings as related to narrative memory, which lies adjacent to but is not fully synonymous with semantic or conceptual similarity. These changes can be found on **Word doc** page 5 line 96 and page 28 line 809; **PDF** pages 5 and 32 lines 96 and 588. We hope this revision clarifies the distinction and addresses your concern.

• P.10, line 204: “PC and PS” these abbreviations were not defined in the article.

**Author response:** Thank you for pointing this out; we have replaced the abbreviations on **Word doc** page 10 lines 210-211; **PDF** page 10 lines 207-208.

• P.9, lines 130-131 or lines 136-137, I suggest citing also the study of Gellersen et al. (2021) to support the authors’ argument.

Gellersen, H. M., Trelle, A. N., Henson, R. N., & Simons, J. S. (2021). Executive function and high ambiguity perceptual discrimination contribute to individual differences in mnemonic discrimination in older adults. Cognition, 209, 104556. https://doi.org/10.1016/j.cognition.2020.104556

**Author response:** Thank you, we have included it on **Word doc** page 6 line 134; **PDF** page 6 line 134.

• p.8, lines 181-182, is the 95% CI of 4.77-6.96 the CI around the t-test? If so, it is surprising that this interval does not include the t value itself (i.e., 10.59). Can the authors double check and correct if needed?

**Author response:** The confidence interval doesn’t contain the t-value because they measure different things. The t = 10.6 is the test statistic that reflects how many standard errors the observed difference in means between the two age groups is away from 0 under the null hypothesis (i.e., no difference) whereas the 95% confidence interval [4.77, 6.96] refers to the range of values for the difference in group means that are plausible based on our sample data. In other words, we are 95% confident that the true difference in Shipley scores between the older and younger groups lies between 4.77 and 6.96 points.

• p.23, lines 481-482 and lines 489-490 seem to repeat the same information (i.e., that VCT performance was not influenced by English language background).

**Author response:** Thank you for pointing this out. We removed one of them on **Word doc** page 25; **PDF** page 29.

• P.27, line 581, it is unclear what “in their ability to recall memories” refers to, especially since there was no proper memory recall task in this study. Also, the sentence lines 583-584 is almost identical to the first sentence of this paragraph (lines 580-581).

**Author response:**

Thank you for raising this point. To clarify, the verbal completion task used in our study is a cued recall task in which participants are explicitly instructed to retrieve the studied word corresponding to a sentence stem. This contrasts with implicit stem-completion tasks where participants complete stems without an explicit memory prompt. Because our task involves intentional retrieval, we believe the description of it as a recall task is appropriate.

That said, we recognize that our original phrasing may have been too general and could have led to misinterpretation. We have revised the text to specify “in their ability to automatically reactivate previously studied items” on **Word doc** page 31, lines 942-943; **PDF** page 37 lines 801-802.

Regarding your second point about redundancy in the paragraph, we agree that the repetition was unnecessary. We have revised the paragraph to eliminate the duplicated content.

We appreciate your careful reading and thoughtful suggestions, which helped us improve both clarity and precision in our discussion.

• p.11, line 248, there seems to be a typo in “Based off of these cues”.

**Author response:** Thank you, we have adjusted it to “Additionally, all words had 3-letter stems that were unique across the whole stimulus set, which served as cues during the retrieval portion of the VCT.” **Word doc** Page 12 lines 318-321; **PDF** page 13 lines 279-281.
