# Supplementary material for: Aging and episodic memory specificity: Evidence challenging a domain-general pattern separation decline
Source: PLoS One. 2025 Nov 24;20(11):e0336045. doi: 10.1371/journal.pone.0336045 (PMC12643304; doi:10.1371/journal.pone.0336045)
Supplement: Attachment — Submitted filename: Response to Reviewers - 10.16.25.docx [file pone.0336045.s007.docx]

**Response to Reviewers**

**Reviewer 1**

Reviewer #1: The authors have addressed my primary concern about d’ floor effects in the VDT task by explaining that the analysis of VDT slope gets around the floor effects. In addition, the lack of a group effect in the slope analysis is consistent with the lack of a group effect in the d’ analysis. I missed this logic in the initial submission, and it is still missing from the revision. Thus, as a naïve reader of the revision, I would still have the same concern about floor effects that I had upon reading the previous version.

As it stands, the semantic similarity analysis of slopes section begins “The analyses above suggest that the foils in the VDT may have had unintended semantic relationships to studied words. To address this,...”

This introduction to the semantic similarity analysis section is still missing the important issue that has arisen in the prior paragraph, where the floor effect d’ analyses are reported. The authors should make it clearer at this stage of reporting that the slope analysis will address the floor effect issue they have just read about.

Following your suggestion, we have modified our section that now reads as: “This conclusion would be premature, however, because behavioral performance suggested that our semantic similarity manipulation was less effective than the object similarity in the MST. Specifically, false alarm rates to semantic foils were higher than those in the MST and closer to lure false alarms in the VDT, reducing our ability to measure the impact of semantic relatedness on false alarm rates and resulting in an outcome akin to a floor effect.

**Semantic similarity results**

The analyses above suggest that the foils in the VDT may have had unintended semantic relationships to studied words. To address this and resolve the issue of the *d′*_a_ floor effect, we leveraged the high variability in false alarm rates across lures and foils to assess more rigorously how semantic similarity influences mnemonic discrimination across age groups.”

Also make it clearer in the Discussion that the findings from the murky d’ analysis that suffers from floor effects is nonetheless concordant with the findings from the slope analysis that does not suffer from floor effects.

Thank you for your suggestion. We have now added this line to our Discussion: “Despite the *d′*_a_ analysis being limited by floor effects, the overall pattern of findings was nonetheless consistent with the results of the slope analysis, which was not subject to the same limitation.”

Finally, given the importance of the slopes for this paper, please report the slope descriptive statistics and p values in Table 2.

Thank you for pointing this out. We added the values to Table 2.

All other concerns have been addressed.

Thank you for your thoughtful points. We really appreciated all of your comments!
